# Region-Specific Reduction of BDNF Protein and Transcripts in the Hippocampus of Juvenile Rats Prenatally Treated With Sodium Valproate

**DOI:** 10.3389/fnmol.2019.00261

**Published:** 2019-11-07

**Authors:** Constanza R. Fuentealba, Jenny L. Fiedler, Francisco A. Peralta, Ana María Avalos, Felipe I. Aguayo, Katherine P. Morgado-Gallardo, Esteban E. Aliaga

**Affiliations:** ^1^Department of Kinesiology, Faculty of Health Sciences, Universidad Católica del Maule, Talca, Chile; ^2^Department of Biochemistry and Molecular Biology, Faculty of Chemical and Pharmaceutical Sciences, Universidad de Chile, Santiago, Chile; ^3^Instituto de Ciencias Biomédicas, Facultad de Ciencias de la Salud, Universidad Autónoma de Chile, Santiago, Chile; ^4^Department of Psychology, Faculty of Health Sciences, Universidad Católica del Maule, Talca, Chile; ^5^The Neuropsychology and Cognitive Neurosciences Research Center (CINPSI-Neurocog), Faculty of Health Sciences, Universidad Católica del Maule, Talca, Chile

**Keywords:** autism, valproate, BDNF, long-3′UTR-bdnf, hippocampus

## Abstract

Autism is a neurodevelopmental disorder characterized by a deep deficit in language and social interaction, accompanied by restricted, stereotyped and repetitive behaviors. The use of genetic autism animal models has revealed that the alteration of the mechanisms controlling the formation and maturation of neural circuits are points of convergence for the physiopathological pathways in several types of autism. Brain Derived Neurotrophic Factor (BDNF), a key multifunctional regulator of brain development, has been related to autism in several ways. However, its precise role is still elusive, in part, due to its extremely complex posttranscriptional regulation. In order to contribute to this topic, we treated prenatal rats with Valproate, a well-validated model of autism, to analyze BDNF levels in the hippocampus of juvenile rats. Valproate-treated rats exhibited an autism-like behavioral profile, characterized by a deficit in social interaction, anxiety-like behavior and repetitive behavior. *In situ* hybridization (ISH) experiments revealed that Valproate reduced BDNF mRNA, especially long-3′UTR-containing transcripts, in specific areas of the dentate gyrus (DG) and CA3 regions. At the same time, Valproate reduced BDNF immunoreactivity in the *suprapyramidal* and *lucidum* layers of CA3, but improved hippocampus-dependent spatial learning. The molecular changes reported here may help to explain the cognitive and behavioral signs of autism and reinforce BDNF as a potential molecular target for this neurodevelopmental disorder.

## Introduction

The Autism Spectrum Disorder (ASD) includes a group of neurodevelopmental disorders. Autism is characterized by deep impairment in social interaction and communication and is always associated to restricted, stereotypic and repetitive behaviors (Frith and Happé, 2005). The biological evidence supports a view in which altered maturation in neural circuitry produces a misconnected brain, characterized by overconnected short distance circuits and poorly connected long-distance circuits (Wass, 2011). This type of functional alteration has been probed in an animal model of autism of prenatal exposition to the anticonvulsant drug Valproate (VPA), where hyper-connectivity, -reactivity and -plasticity have been electrophysiologically evidenced in local circuits of amygdala and cortical areas (Markram et al., 2008; Rinaldi et al., 2008a,b; Silva et al., 2009).

The current higher rates of autism diagnosis strongly suggest that environmental factors, in concert with genetic risk factors, cause this developmental disorder (Schaaf and Zoghbi, 2011). The genes related to ASD traits include postsynaptic cell adhesion and scaffold synaptic proteins (Jamain et al., 2003; Durand et al., 2007). However, not only structural synaptic proteins are involved, as shown by Angelman and Rett syndromes, both of which comprise part of the spectrum of neurologic disorders associated with autism. The mutation produced in the UBE3A ligase gene in Angelman syndrome, for example, revealed that the turnover of synaptic proteins might also be a key process in the developmental plasticity underlying neural circuit maturation (Kishino et al., 1997; Dindot et al., 2008). In the Rett syndrome, a mutation in the transcriptional repressor MeCP2 (methylated-cytosine binding protein) alters the expression of several genes, including Brain-Derived Neurotrophic Factor (BDNF), where its dysregulation seems to play a critical role in the neurobiological consequences of this syndrome (Li and Pozzo-Miller, 2014). In fact, other genes involved in ASD are neurotrophin release-related proteins, such as Calcium Dependent Activator Protein, CAPS2, suggesting a role for these types of critical regulators of neuronal development in ASD (De Rubeis et al., 2014).

BDNF plays multiple roles in nervous system development and plasticity (Kowiański et al., 2018) and increasing evidence in humans supports its role in different types of autism. Medication-free ASD children show high levels of serum BDNF and increased BDNF mRNA in lymphocytes (Miyazaki et al., 2004; Connolly et al., 2006; Nishimura et al., 2007; Meng et al., 2017). Interestingly, BDNF levels have also been associated with ASD-like traits in the general population (Brondino et al., 2018), and recent meta-analysis studies suggest that BDNF levels may be considered as a possible biomarker for autism (Qin et al., 2016; Zheng et al., 2016; Armeanu et al., 2017; Saghazadeh and Rezaei, 2017). However, in spite of the abundant literature that relates BDNF levels with autism in different ways, the precise role of BDNF in this type of disorder remains unclear (Rahmani and Rezaei, 2017).

The best-validated experimental model of induced-autism has emerged from the clinical correlation between prenatal exposure to VPA and autism prevalence (Alsdorf and Wyszynski, 2005). In the prenatal exposure to VPA model of autism (VPA-model), treatment of pregnant rats with a single dose of VPA during a narrow window of embryonic period (around E12.5) produces a behavioral syndrome that resembles autism (Wagner et al., 2006). Evidence from the VPA-model indicates that there is an acute increase in BDNF expression in the mouse embryonic brain; mRNA levels reach a maximum at 3 h and protein levels reach their highest value after 6 h of VPA administration (Almeida et al., 2014; Hara et al., 2017).

In contrast to the acute effect on BDNF levels, the postnatal effect of embryonic VPA treatment is more diverse and questionable. For instance, some reports in the hippocampus have shown no change, reduction or increase in BDNF mRNA levels (Yamaguchi et al., 2017; Win-Shwe et al., 2018), while it increases in the somatosensory cortex (Roullet et al., 2010). The prenatal VPA-model of autism also shows disparities in BDNF protein levels, i.e., they increase in the hippocampus (Gao et al., 2016; Rahimi et al., 2018), but decrease in the cortex (Chau et al., 2017). However, BDNF protein levels have always been studied using whole tissue homogenates, which impedes the observation of regional differences.

BDNF gene regulation is extremely complex, generating different transcripts and different peptide isoforms with distinctive biological functions (Kowiański et al., 2018). In particular, the mammalian BDNF gene can generate two families of transcripts: one containing short-3′UTR (S-*bdnf*), and another one characterized by long-3′UTR (L-*bdnf*; Timmusk et al., 1993; Liu et al., 2006; Aid et al., 2007; Pruunsild et al., 2007). Both *S-bdnf* and* L-bdnf* transcripts contain the complete coding sequence, and their translation generates a pre-proBDNF. Intracellularly, this precursor must be cleaved to produce proBDNF, which must be further processed to produce the mature form (mBDNF), which is secreted in an activity-dependent way and interacts with the TrkB receptor (Greenberg et al., 2009). However, under some conditions, proBDNF can also be secreted and then interact with the p75 neurotrophin receptor, determining that mBDNF has opposing effects in neural survival, development and plasticity (Kowiański et al., 2018). Even though S-*bdnf*, and L-*bdnf* can be found in somatic and dendritic compartments, it is accepted that L-*bdnf* transcripts are targeted to dendrites and can be locally translated in response to synaptic activity (An et al., 2008). Interestingly, reports indicate that somatically or dendritically synthesized BDNF can have differential roles on dendritic spine morphogenesis in cultured cortical neurons. In fact, proBDNF produced by local synaptic translation from the L*-bdnf* transcript can be secreted to promote synaptic pruning, probably through p75 signaling, while processing of proBDNF by extracellular proteases to generate mBDNF, promotes synaptic maturation (Orefice et al., 2013, 2016). Interestingly, excessive dendritic targeting of BDNF transcripts in the hippocampus has been related to epileptogenesis (Simonato et al., 2002; Tongiorgi et al., 2006), while the absence of L-*bdnf* produces deficient synaptic pruning and blunted synaptic plasticity (An et al., 2008). Of note, there is a high prevalence of epilepsy co-occurring with autism (Muñoz-Yunta et al., 2008) and deficient synaptic pruning has been proposed as a core cellular defect in autism (Tang et al., 2014).

According to the previous evidence, differentiating the type of BDNF transcripts (total or *L-bdnf*) and the precise place where changes in transcripts and protein levels occur in early postnatal age after embryonic VPA treatment may contribute to understanding the role of BDNF in the deviated neurodevelopmental trajectory of autism. Therefore, our goal was to determine BDNF immunoreactivity (BDNF-IR) and mRNA levels (total and L*-bdnf* transcripts) in different subregions of the hippocampus in juvenile rats (postnatal day 30, PN30), prenatally treated with VPA. In the VPA-treated rats, which showed autism-like behavior, we found reduced levels of BDNF mRNA (especially L*-bdnf)* in the dentate gyrus (DG) and CA3 areas. In addition, BDNF-IR was reduced in dendritic layers of CA3 and the suprapyramidal and lucidum layers. Furthermore, experimental rats showed increased spatial memory, expressed as a rise of the memory index in the Y-maze test. These results support the idea that, in contrast to the acute upregulating effect of VPA administration in fetal brain BDNF, a downregulating effect occurs in specific regions of the hippocampus at the juvenile stage. Therefore, an adaptive long-time reduction of BDNF levels may be proposed as a response to the acute increase that occurs during early stages of brain development.

## Materials and Methods

### Animals

Sprague–Dawley rats were maintained under standard conditions (21°C; 12/12 light-dark cycle) with *ad libitum* access to food and water. At the embryonic day 12.5 (E12.5), pregnant rats were injected with a single dose of Sodium Valproate dissolved in saline (450 mg/Kg i.p. Sigma-Aldrich, St. Louis, MO, USA), while the control group received an injection of saline solution. On the day of weaning, at postnatal day 21 (PN21), males were separated from their dam and housed in groups of 3–4 littermates.

Only males were used in this work, considering the higher prevalence of autism in males (American Association Psychiatric, 2013; Loomes et al., 2017), even though prenatal VPA treatment produces effects both in males and females but, in general, a less intense effect in females (Hara et al., 2017; Al Sagheer et al., 2018). All efforts were made to minimize the number and the suffering of animals and procedures were approved by the Ethic Committee of the Chilean Science and Technology National Commission (CONICYT), in compliance with the National Institutes of Health Guide for Care and Use of Laboratory Animals (NIH Publication, 8th Edition, 2011).

### Behavioral Tests

Social behavior and anxiety were analyzed at PN30 using the three-chamber social test and elevated plus maze test, respectively; as we have previously shown (Peralta et al., 2016). For the social test, total interaction time was 10 min and control and experimental animals were exposed to a familiar congener located in one of the lateral chambers inside of a wire cage, while the other chamber remained empty. The time spent in the occupied chamber was considered as “time in social area” and the time at the center chamber plus the time in the empty chamber was considered as “time in no social area.” As an additional measure of social interaction, total time spent by the tested animal sniffing the stimulus cage in the social chamber (touching with or introducing the nose in the wire cage) was determined from the social test videos by an operator blind to the condition of the animal. In the case of the elevated plus maze, total exploration time was 5 min. All tests were conducted between 9:00 and 15:00 h, using 60 db white noise and the apparatus was cleaned after each test. Both tests were filmed using a superior view and analyzed by a blind operator with the ANY-maze software (Stöelting, Wood Dale, IL, USA). As an indicator of stereotypic behavior, the number of events and the time spent in grooming activity was determined from the social test videos by a trained operator.

### Preparation of Riboprobes

The riboprobe to detect all BDNF transcripts (also named panBDNF) was prepared using a 186-bp fragment (325–508 bp, GenBank: M61175) of coding exon (IX according to Aid et al., 2007), previously cloned in the pGEMT vector and validated for *in situ* hybridization (ISH) by our group (Aliaga et al., 2009). The plasmid was linearized and the antisense digoxigenin-labeled riboprobe (DIG-riboprobe) was transcribed using T7 RNA polymerase (Roche, Indianapolis, IN, USA). The detection of BDNF transcripts specifically containing the 3′UTR-long region (*L-bdnf*) was carried out by polymerase chain reaction (PCR)-cloning of specific fragments of each corresponding gene, from cDNA obtained by retrotranscription of total RNA from adult rat hippocampus. A 213-pb fragment of the BDNF gene located between the two alternative polyadenylation sites in the 3′ extreme of exon IX (2917–3129 pb, GenBank: X67108) was amplified (forward primer: 5′-gccacctggaagagtacctg-3′, reverse primer: 5′-ggaactggtcaagtggctca-3′) and inserted in the pGEMTeasy vector. The vector was linearized and SP6 RNA polymerase was used to obtain antisense DIG-riboprobe by *in vitro* transcription (Roche, Indianapolis, IN, USA).

### Non-isotopic *in situ* Hybridization

Twenty-four hours after the last behavioral test, a deep anesthesia was induced by Isoflurane (Baxter), and animals were euthanized by decapitation. Whole brain was quickly extracted, divided into three blocks, fixed overnight in 4% p-formaldehyde, and stored in 30% sucrose phosphate-buffered saline solution (PBS). ISH studies using DIG-riboprobes were performed using the standard free-floating protocol (Aliaga et al., 2009). Briefly, coronal sections (40 μm) of control and experimental brains containing dorsal hippocampus were cut off on a MICROM HM525 cryostat (Thermo Scientific, Waltham, MA, USA) and collected in room temperature PBS. The sections were rinsed three times in PBS and incubated for 1 h at 50°C in 4× SSC (saline-sodium citrate: 20× SSC is 3 M sodium chloride and 300 mM trisodium citrate, pH 7.0), containing 1× Denhardt’s solution and 50% formamide. After prehybridization, sections were incubated overnight at 50°C with denatured DIG-riboprobe (100 ng/ml) in hybridization solution (4× SSC, 1× Denhardt’s solution, 50% formamide, 2 mM EDTA, 0.1% SDS, 1 mM DTT, 100 μg/ml salmon sperm DNA and 0.2% sarcosyl). Post-hybridization treatment in decreasing ionic strength included 10 min rinses in 4×, 2×, 1× and 0.5× SSC at room temperature, and finally 1 h in 0.1× SSC at 50°C. Immunohistochemical label development by alkaline phosphatase-conjugated anti-DIG antibody was conducted as previously described (Aliaga et al., 2002) and visualized by incubation with 4-nitro-blue-tetrazolium and 5-bromo-4-chloro-3-indolyl-phosphate (Roche, Indianapolis, IN, USA). Negative controls were carried out on similar sections with sense riboprobes for each of the three riboprobes and did not show significant staining (not shown).

### Spatial Memory Test

Spatial memory was evaluated with the Y-maze, as described (Conrad et al., 2003). This maze consists of three 46 × 13 × 32 cm arms arranged at 120 degrees between them. In the first stage, one animal explores only two arms of the maze for 15 min and is then returned to its home cage. In a second stage, 2 h later, the animal explores the three arms during a 5 min period. Preference for the new arm is considered as an index of memory (Conrad et al., 1996). As another indicator of stereotypic behavior, the number of re-entries in the same arm was determined from the Y-maze videos by a trained operator.

### Immunohistochemistry

Coronal sections equivalent to those used in the ISH protocol were destined for BDNF immunoreactivity detection by free-floating Immunohistochemistry (IHC), using a previously described procedure (Peralta et al., 2016). Briefly, endogenous peroxidase was blocked by incubation with 0.3% H_2_O_2_ during 45 min, and sections were incubated overnight at room temperature with primary antibody (1:100 polyclonal anti-BDNF, Santa Cruz, CA, USA) in blocking solution (3% BSA, 0.4% Triton X-100 PBS). On the second day, immunostaining was developed using 1:1,000 biotinylated secondary anti-mouse antibody (Jackson ImmunoResearch Labs, West Grove, PA, USA) and the avidin/biotin/peroxidase complex amplification system with DAB Peroxidase Substrate, both from Vector Laboratories (Burlingame, CA, USA). Since the anti-BDNF antibody is a commercial product, negative control was performed by incubation without primary antibody, and no label was found (not shown).

### Image Analysis

In order to quantify the intensity of ISH and IHC labels in the different hippocampal subfields, digital microphotographs were taken with a Carl Zeiss Axiolab E microscope, using a Cannon EOS Rebel T3 digital camera. Photographs (5×) of the hippocampus were used to determine the *in situ* labeling by using the “mean intensity” function of the ImageJ software (National Health Institute, Bethesda, MD, USA). A polygonal region of interest, “ROI,” was defined for each measured region and mean intensity value was obtained. This value was corrected by subtracting the background, determined for each particular tissue section and was always obtained as the mean of three cell-free zones (excluding possible glial stained-cells) of the corpus callosum or fimbria. In addition, we discarded CA2 (between CA1 and CA3a), and a zone between CA3a and CA3b, in order to avoid any errors of anatomical assignment in these two transitional zones. We verified that the mean background value for ISH was not statistically different from the intensity value of negative controls, performed with a sense riboprobe (*p* = 0.76, *t*-test). The same was observed for IHC, where negative controls performed without the primary antibody showed no differences in mean intensity compared to mean background levels of stained sections (*p* = 0.34, *t*-test). This procedure was applied to each tissue section, as we (Aliaga et al., 2002; Bravo et al., 2006), and other groups have previously reported (Chiaruttini et al., 2008; Sampathkumar et al., 2016; Rocha et al., 2017). For each region, the measures were taken from 7 to 8 (ISH) or 7 (IHC) independent animals, and each value represents the intensity average of two or three slices from the same animal. For each determination, all the samples were processed on the same day and standardized as percentage of the control.

### Statistical Analysis

Statistical analyses were performed using GraphPad Prism 6.0 (GraphPad Software Inc., San Diego, CA, USA). All data obtained from the behavioral tests and histological determinations were expressed as mean ± standard error of the mean (SEM) and were processed for normality test distribution (Kolmogorov–Smirnov test). When data showed normality, statistical significance was assayed with the two-tailed Student’s *t*-test. In the cases where no normality was found, the non-parametric two-tailed Mann–Whitney *U*-test was used. In all cases, a *p*-value lower than 0.05 was considered as significant.

## Results

**Figure 1:**
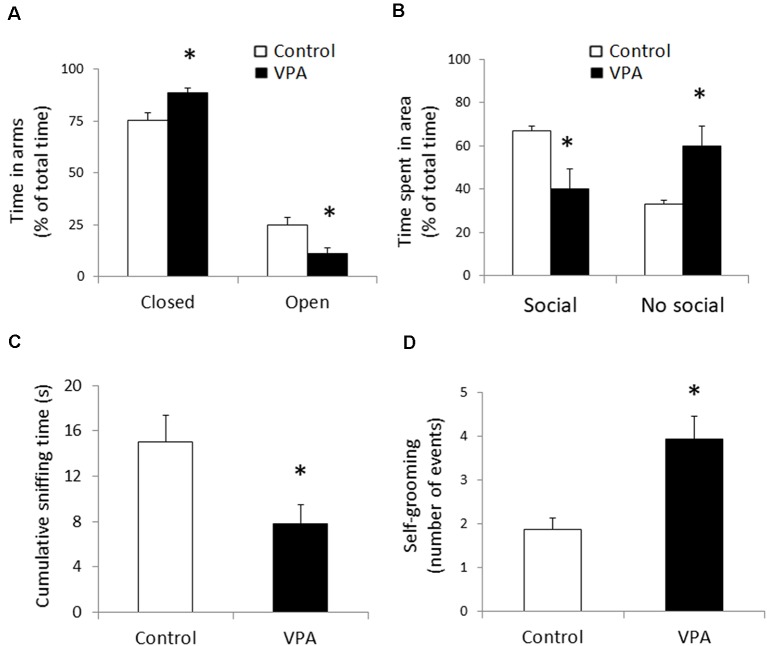
Prenatal Valproate treatment produces autism-like behavior. Percentage of the total time spent in closed and open arms in the elevated plus maze** (A)**. Percentage of the total time spent in social and non-social areas in the three-chamber social test **(B)**. Cumulative sniffing time in social chamber of the three-chamber social test **(C)**. Number of self-grooming events during the social test **(D)**. The data represent the mean ± standard error of the mean (SEM) and controls are represented by white bars, while VPA-treated animals are represented by black bars. *p < 0.05 *t*-test (*n* = 10) for elevated plus maze and *p < 0.05 *U*-test (*n* = 8) for all parameters from the social test.

First, we verified if prenatal VPA-treatment triggers the three most important behavioral signs observed in ASD: anxiety-like behavior, social deficit, and stereotypic behaviors. In order to detect anxiety-like behavior, we used the elevated plus maze and we found a statistically significant increase in the accumulated time spent in the closed arms, expressed as a percentage of the total time of the test (88.74 ± 2.48% in VPA vs. 75.31 ± 3.62% in control, *p* = 0.0036, *U* = 13, Mann–Whitney test, *n* = 10). Conversely, accumulated time spent in the open arms decreased (11.3 ± 2.48% in VPA vs. 24.69 ± 3.62% in control, *p* = 0.0036, *U* = 13, Mann–Whitney test, *n* = 10; Figure 1A). This result shows that, in our hands, the VPA-model induces anxiety-like behavior.

With the idea of evaluating changes in social interaction in the VPA-model, we used the social preference protocol in the three-chamber social test, using a known congener in one of the lateral chambers, while the other one remained empty. In contrast with the control group that displayed a strong preference for spending time in the social area, the VPA group showed a reduced preference for that area (67.1 ± 2.1% of total time for control vs. 40.2 ± 9.2% for VPA, *p* = 0.0083, *t* = 3.071, *df* = 14, unpaired *t*-test, *n* = 8). On the other hand, the VPA group showed a higher preference for the non-social area, compared to the control group (59.9 ± 9.2% for VPA vs. 32.9 ± 2.1 for the control group, *p* = 0.0083, *t* = 3.071, *df* = 14, unpaired *t*-test, *n* = 8; Figure 1B). Additionally, to strengthen our measure of sociability, we evaluated sniffing behavior of the tested animal around the social stimulus, encaged in the social chamber, quantifying the cumulative sniffing time. VPA-treatment produced a statistically significant reduction in the sniffing time (14.99 ± 2.42 s for control vs. 7.8 ± 1.67 s for VPA, *p* = 0.027, *U* = 11, Mann–Whitney test, *n* = 8), evidencing a social deficit effect in the experimental group (Figure 1C). The loss of social preference and the reduction in sniffing time support a social deficit in our VPA group.

In order to evaluate stereotypic behavior, we quantified the number of self-grooming events during the whole duration of the social test (Figure 1D). Compared to the controls, that showed a number of self-grooming events of 1.9 ± 0.3; VPA treatment doubled that quantity (3.9 ± 0.5 events in 10 min) in a statistically significant way (*p* = 0.0006, *U* = 1.1, Mann–Whitney test, *n* = 8). In contrast, the duration of each self-grooming event did not show a significant variation (7.2 ± 1.4 s for the VPA group, compared to 5.7 ± 1.9 s in the controls). These data suggest that, in our experimental animals, VPA-treatment triggers one of the most robust hallmarks of autism; i.e., a stereotypic behavior.

**Figure 2:**
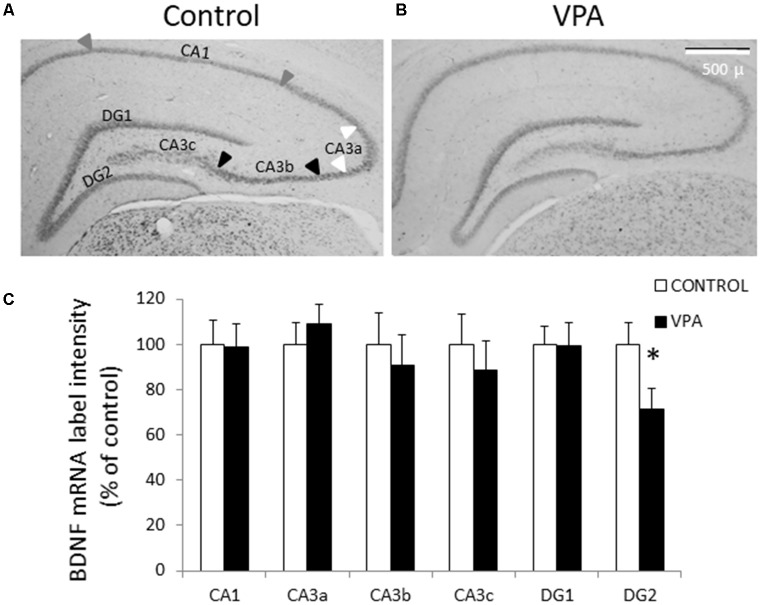
Prenatal VPA treatment decreases brain derived neurotrophic factor (BDNF) mRNA levels in the inferior blade of the dentate gyrus (DG) hippocampal region. Representative microphotographs showing *in situ* hybridization (ISH) labeling for pan-*bdnf* transcripts in postnatal day 30 (PN30) hippocampus from controls **(A)** and prenatally VPA-treated rats **(B)**. Immunolabeling intensity of digital microphotographs from control and experimental animals was analyzed in the different layers of hippocampal subfields and expressed as percentage of controls **(C)**. The data represent the mean ± SEM of BDNF mRNA label intensity. Controls are represented by white bars and VPA-treated animals are represented by black bars. *p < 0.05, *U*-test. *n* = 8 for control and VPA-treated rats. DG1: superior blade of DG, DG2: inferior blade of DG, CA1: *Cornu ammonis* region 1, CA3a-c: *Cornu ammonis* region 3 from the semicircular lateral extreme (CA3a) to the polymorphic zone entering in the hilus (CA3c), with the horizontal pyramidal zone (CA3b) between these two regions. Arrowheads in **(A)** indicates the CA1 (gray), CA3a (white) and CA3b (black) regions considered for the analysis. The scale bar represents 500 μm.

Diverse evidences indicate that different types of autism present disturbances in neuronal development and synaptic plasticity, probably due to a dysfunction of neurotrophic factors, particularly BDNF. To detect BDNF mRNA, we first conducted ISH using a Digoxigenin-labeled riboprobe complementary to the IX-exon of the BDNF gene, which allowed us to detect all types of BDNF transcripts (also named *pan-bdnf*). Prenatal VPA treatment produced a selective and statistically significant decline in *pan*-*bdnf* transcripts in the inferior (or ventral) blade of the DG granular cell layer (DG2 in Figure 2), but not in the superior blade of the DG (DG1). In fact, while the control group showed a standardized intensity label of 100 ± 9.6% in GD2, the values of the VPA group decreased to 71.3 ± 9.3% (*p* = 0.0281, *U* = 1.1, Mann–Whitney test, *n* = 8).

**Figure 3:**
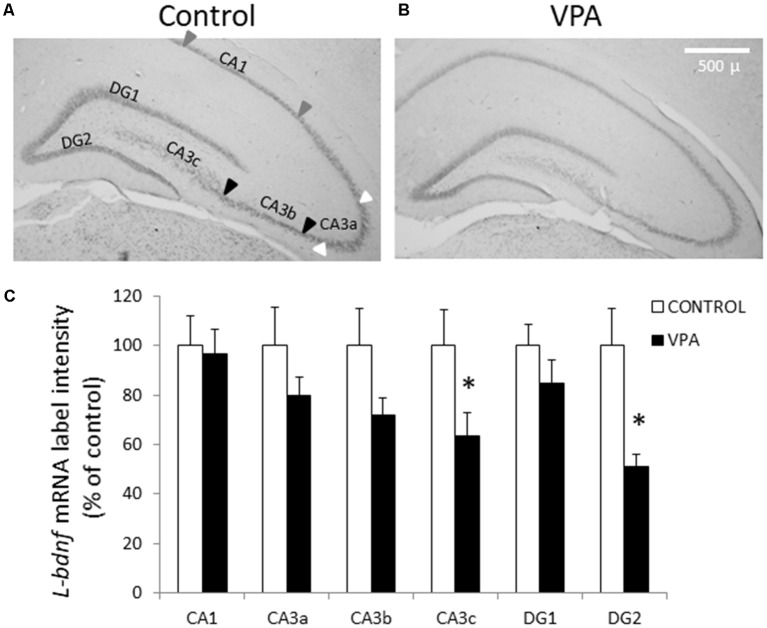
Prenatal VPA treatment decreases *L-bdnf* mRNA level in the inferior blade of DG and in the polymorphic layer of the CA3 hippocampal region. Representative microphotographs showing ISH labeling, using a specific riboprobe to detect *L-bdnf* transcripts in PN30 hippocampus from controls **(A)** and prenatally VPA-treated rats **(B)**. ISH label intensity of digital microphotographs from control and experimental animals was analyzed in the different layers of hippocampal subfields and expressed as percentage of controls **(C)**. The data represent the mean ± SEM of *L-bdnf* mRNA label intensity. Controls are represented by white bars and VPA-treated animals are represented by black bars. *p < 0.05, *U*-test. *n* = 8 for control and VPA-treated rats. Scale bar, arrowheads and hippocampal areas are the same as described in Figure 2.

To obtain further insight into the effect of prenatal VPA treatment on BDNF transcript levels, we conducted ISH experiments to detect *L-bdnf* transcripts using a digoxigenin-labeled riboprobe complementary to a sequence located between the two alternative polyadenylation sites on the 3′UTR extremes. In contrast to *pan-bdnf ISH* label, where the only effect was a specific reduction in the GD2 layer, *L-bdnf ISH* label showed a more general tendency to reduce its intensity in the DG and CA3 areas, with statistically significant effects in both CA3c and DG2 regions (Figure 3). While control groups showed a normalized label intensity of 100 ± 15%, the intensity of the VPA group decreased to 51 ± 5.1% in GD2 (*p* = 0.003, *U* = 5, Mann–Whitney test, *n* = 8). In CA3c—the polymorphic zone of CA3 located inside of the DG hilus—standardized label intensity of controls was 100 ± 14.7%, while VPA treatment decreased intensity values to 63.7 ± 9.3% (*p* = 0.0499, *U* = 13, Mann–Whitney test, *n* = 8).

**Figure 4:**
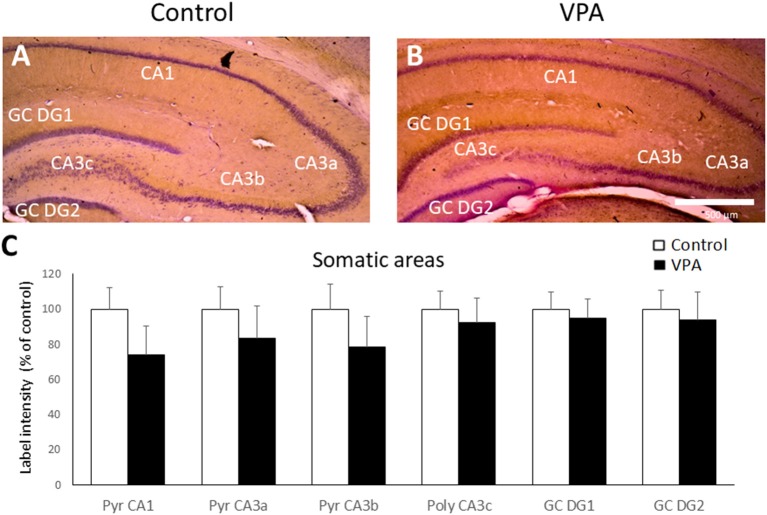
Prenatal VPA treatment produces no effect on BDNF-IR in somatic layers of the hippocampus. Representative microphotographs of Cresyl violet counterstained BDNF Immunohistochemistry (IHC) of controls **(A)** and VPA-treated **(B)** rats. Higher magnification of single stained slices was used to quantify the IR label intensity in each hippocampal region **(C)**. Data represent the mean ± SEM. White and black bars represent controls and VPA-treated animals, respectively. *n* = 7 for control and VPA-treated rats. Scale bar represents 500 μm. GC DG1: superior blade of granule cell layer of DG, GC DG2: inferior blade of granule cell layer of DG, Pyr: *stratum pyramidale*, CA1: *cornu ammonis* region 1, CA3a-c: *cornu ammonis* regions 3 a to c.

We then evaluated BDNF peptide levels in the hippocampus of control and VPA-treated rats by IHC. BDNF-IR was found in all the cellular layers of the hippocampus, including the granular cell layer of DG, the pyramidal layer of CA1 and CA3, and the polymorphic region of CA3 (CA3c). As a reference, Cresyl violet counterstained BDNF IHC of control and VPA-treated rats is shown in Figures 4A,B. Higher magnification (20 or 40×) microphotography of single stained samples (not counterstained) were used to quantify BDNF-IR label intensity in each hippocampal somatic layer. As is shown in Figure 4C, VPA treatment did not produce any effect on BDNF-IR in somatic layers of the hippocampus.

**Figure 5:**
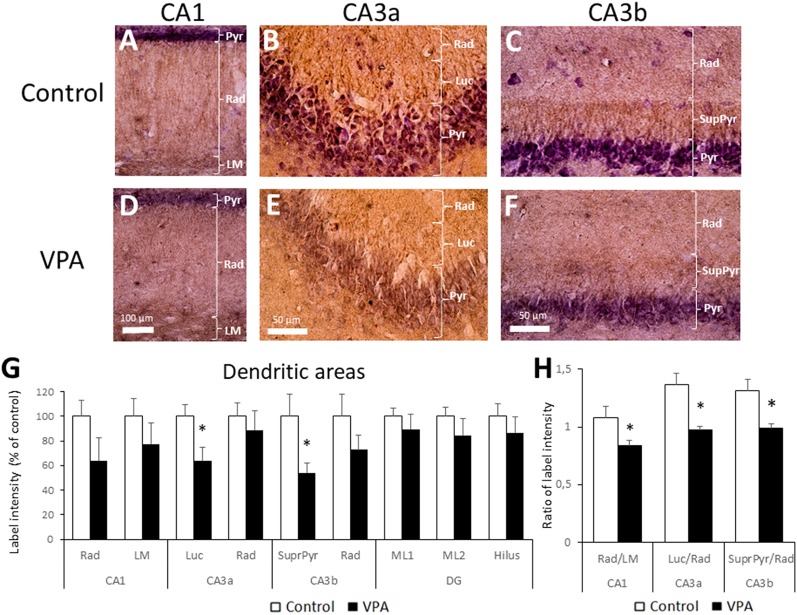
Prenatal VPA treatment decreases BDNF inmunoreactivity in the CA3 *lucidum* and suprapyramidal layers. Representative microphotographs of CA1 **(A,D)**, CA3a **(B,E)** and CA3b **(D,F)** of control **(A–C)** and VPA-treated **(D–F)** rats. LM: *stratum lacunosum moleculare*, Rad: *Stratum radiatum*, Luc: *Stratum lucidum* and SupraPyr: suprapyramidal layer. The bar represents 500 μm. **(G)** Bar graph representing the label intensity in dendritic areas of the hippocampus and DG. **(H)** Ratio of label intensity between proximal vs. distal portion of dendritic regions in CA1, CA3a and CA3b. The data represent the mean ± SEM. Controls are represented by white bars and VPA treated animals are represented by black bars. *p < 0.05, *t*-test, except for SupraPyr/Rad ratio, where *U*-test was used. *n* = 7 for control and VPA-treated rats in all cases.

The dendritic layer (also named neuropil) showed BDNF-IR staining as well, including the CA1 and CA3 *stratum radiatum*, CA1 *lacunosum moleculare* (LM), CA3 *stratum lucidum*, and CA3 suprapyramidal layers. The latter corresponds to the proximal dendritic layer immediately over the CA3b pyramidal layer. More prominent BDNF-IR was found in the CA1 LM and in *lucidum* and suprapyramidal layers of the CA3 region (Figures 5A–F). We used higher magnification of single stained samples to quantify BDNF-IR intensity in each hippocampal region and observed a statistically significant decrease in the *stratum lucidum* of CA3a and in the suprapyramidal layer of CA3b (Figure 5G). While control rats showed a standardized BDNF-IR of 100 ± 9.75% in CA3a* stratum lucidum*, label intensities of VPA groups dropped to 64.1 ± 10.8% (*p* = 0.0202, *t* = 2.676, *df* = 12, unpaired *t*-test, *n* = 7). On the other hand, control levels (100 ± 17.9%) in the CA3b suprapyramidal layer decreased to 54.1 ± 7.7% in the VPA group (*p* = 0.0257, *t* = 2.546, *df* = 12, unpaired *t-test*, *n* = 7).

In order to express BDNF-IR differences between adjacent dendritic layers; e.g., *radiatum* vs. LM layers in CA1, or *lucidum* and suprapyramidal layers in relation to *radiatum* in CA3a and CA3b, respectively, we calculated the ratio of the intensity between adjacent neuropil layers. The comparison of BDNF-IR in these three pairs of dendritic zones showed statistical differences. The *radiatum*/LM ratio in CA1 in the control group (1.08 ± 0.08) was reduced in the VPA group (0.84 ± 0.04; *p* = 0.0125, *t* = 2.936, *df* = 12, unpaired *t*-test, *n* = 7). Similarly, the *lucidum*/*radiatum* ratio in CA3a of controls (1.37 ± 0.13) was reduced in VPA (0.98 ± 0.02) in a statistically significant way (*p* = 0.0065, *t* = 3.283, *df* = 12, unpaired *t*-test, *n* = 7). Finally, the suprapyramidal/*radiatum* ratio in CA3b was also reduced by prenatal VPA treatment (1.31 ± 0.1 in control vs. 0.99 ± 0.04 in the VPA group, *p* = 0.0035, *U* = 3, Mann–Whitney test, *n* = 7; Figure 5H). Noteworthy, in all cases, the reduction in the BDNF-IR ratio involves a superior BDNF-IR in distal dendritic regions, relative to the proximal dendritic regions in VPA rats.

**Figure 6:**
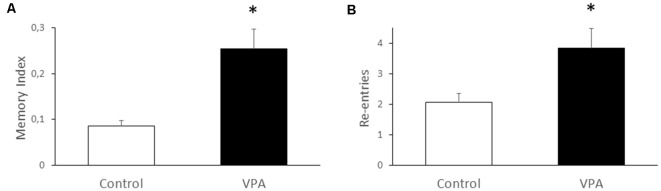
Effect of prenatal VPA treatment on spatial memory and repetitive behavior in the Y-maze. Prenatal VPA treatment improves spatial memory in Y-maze test **(A)**. Spatial memory was evaluated by the permanence index, expressing the proportion of the time spent in the novel arm, in relation to the time spent in the novel plus the known arm. The number of reentries in the same arm was evaluated as a repetitive behavior during the Y-maze test **(B)**. The data are expressed as the mean ± SEM. *p < 0.05, *U*-test. *n* = 13 for controls and *n* = 5 for VPA-treated rats.

Since the most studied function of the hippocampus is spatial memory, we used the Y-maze test to evaluate if prenatal VPA-treatment alters this type of memory. We found that VPA-treatment produces nearly a two-fold increase of the memory index, expressed as the time exploring the novel arm, in relation to total time exploring the novel and known arms, after an interval of 2 h between the first and second trial (0.085 ± 0.012 for control vs. 0.254 ± 0.04 for VPA, *p* = 0.0079, *U* = 0, Mann–Whitney test, *n* = 5; Figure 6A). At the same time, we analyzed the re-entries in the same arms as an index of the repetitive behavior trait in VPA-treated rats (2.067 ± 0.28 for control vs. 3.833 ± 0.74 for VPA, *p* = 0.011, *U* = 15, Mann–Whitney test, *n* = 5 for control and 15 for VPA; Figure 6B). These data suggest an increased spatial memory, even in the presence of repetitive behaviors.

## Discussion

Several studies, including our recent report (Peralta et al., 2016), have indicated that treatment of rats with a single dose of sodium valproate at gestational day 12.5 triggers some behavioral alterations which resemble those observed in autism; i.e., social deficit, anxiety and repetitive behaviors. In the present study, and using the VPA-model in Sprague–Dawley rats, we have found autism-like behaviors that were associated with a reduction in BDNF transcripts containing the long-3′UTR in the inferior blade of the DG and in the polymorphic layer of the CA3 region. These modifications were concomitant with a reduction in BDNF-IR in hippocampal areas corresponding to the suprapyramidal zone of CA3 and *stratum lucidum*, both areas targeted by mossy fibers from dentate granule cells.

This is the first study in the VPA-model that demonstrates a reduction in *L-bdnf* transcripts, which are known to be locally translated, generating pro-BDNF, a peptide that binds and transduces through p75 signaling, and that has opposing effects to mBDNF (Kowiański et al., 2018). The functional significance of these findings is that in this VPA-model, there may be an adjustment in mature vs. pro-BDNF production at the hippocampus; changes that may explain the enhanced spatial memory observed in the Y-maze test.

In the present study, we assessed the long-term effect of embryonic exposure to VPA on the performance in specific behavioral tasks at a juvenile stage. At PN30, we observed anxiety-like behavior in the elevated plus maze; change that has been described in this model (Markram et al., 2008) and in ASD patients (McVey, 2019). In relation to social interaction, VPA-animals did not discriminate between social and non-social areas, suggesting an alteration in the motivational component of social behavior, rather than social aversion, finding which is in line with the “Social Motivation Theory of Autism” (Chevallier et al., 2012). Similar to our previous findings (Peralta et al., 2016), in the present study, we also found increased stereotypic movements in the VPA-group. In our prior study, we observed increased stereotypic movements during the open field test and in the present study, we also observed stereotypic movements during the social test. Notably, these antecedents indicate that stereotypic behavior in the VPA-model of autism can be observed independently of the testing paradigm. In addition, in the present study, we observed that the mean time of each self-grooming event was similar between control and experimental groups, suggesting that early VPA treatment affects the recurrence of behavior. These data, in global, support the idea that prenatal VPA administration produces changes in brain neurocircuitry, triggering the three most important hallmark signs of autism, validating our studies.

BDNF is a key neurotrophin that acts as a multifunctional regulator of the formation and maturation of neural circuits. Interestingly, activity-dependent refinement of brain circuitry by synaptic pruning is a developmental process that has been critically compromised in autism (LeBlanc and Fagiolini, 2011; Tang et al., 2014). Considering that we were interested in the early events related to the emergence of ASD-signs, we studied BDNF transcripts and protein levels at the juvenile stage in the VPA-model. Previous evidence indicates that VPA exposure during the embryonic stage (E12.5) produces an acute and transient effect on BDNF expression in the mouse, where BDNF mRNA levels reach a maximum at 2–3 h, followed by a rise in BDNF peptide after 6 h post-VPA injection (Almeida et al., 2014; Hara et al., 2017). Nevertheless, the long-term effect of embryonic exposure to VPA on BDNF at postnatal age has been poorly documented. Previously, two reports have evaluated the variations in BDNF mRNA levels associated to the translated exon by RT-PCR in the hippocampus after E12.5 VPA administration in rodents. One report showed reduced levels of BDNF mRNA in whole hippocampus of male rats at PN90, with no changes in female rats (Win-Shwe et al., 2018). However, the other study used a similar approach and showed no changes in PN60 mice (Yamaguchi et al., 2017). The disparities in these reports may be related to the different age and species used. Moreover, some studies have used ISH with radioactive probes to detect all BDNF mRNA isoforms at PN60 in mice prenatally treated with VPA (E11), and revealed no change in the hippocampus, but an increase in the S1 somatosensory cortex (Roullet et al., 2010). ISH experiments conducted with BTBR T+tf/J (BTBR) mice, an inbred strain model of autism, revealed a specific reduction of BDNF mRNA, reaching 73% in DG and 65% in CA3 (Stephenson et al., 2011). In the present study, we used a *pan-bdnf* riboprobe that detects all isoforms of BDNF transcripts, and we found a specific reduction of label in the inferior blade of DG. Furthermore, using a riboprobe designed to detect *L-bdnf*, we found a significant reduction in the inferior blade of DG and in the polymorphic layer of CA3. Noteworthy, both regions are very close to each other and both receive afferences from DG through the mossy fibers.

We also evaluated BDNF peptide levels by IHC in somatic and dendritic layers, finding no differences in somatic layers between control and VPA-animals. However, we found a significant reduction of BDNF-IR in two dendritic areas: *lucidum* and suprapyramidal regions in CA3a and CA3b, respectively. These region-specific changes in dendritic fields observed in our study may help to explain the heterogeneity of reported data about the effect of prenatal VPA on BDNF protein levels in the hippocampus. To the best of our knowledge, until this report, the evaluation of BDNF protein in the VPA-model of autism has been performed using total extracts of hippocampus and Western-blot or Elisa techniques. The only previous work that evaluated the long-term effect of VPA-treatment (E12.5), but at PN40 in Wistar rats, showed increased BDNF peptide in whole hippocampus extract by Western-blot (Gao et al., 2016). Low doses but extended prenatal VPA treatment (from E7 to E18), that also trigger autism-like behaviors, produced an increase of BDNF levels in the hippocampus of PN30 Wistar rats, as detected by Elisa assay (Rahimi et al., 2018). Furthermore, postnatal treatment with VPA in Sprague–Dawley rats, also proposed as a model for autism-like syndrome (Wagner et al., 2006), reduced hippocampal BDNF levels according to western-blot analysis; with either one injection in PN14 (Lee et al., 2016) or repeated injections from PN10 to PN30 (Welbat et al., 2016). In consequence, our work is not directly comparable with the previous reports on the field, but it is the first study showing BDNF-IR in hippocampal slices, where regional distribution can be analyzed. A statistically significant reduction of intensity ratio between adjacent dendritic layers was found in three hippocampal regions. In particular, BDNF-IR reduction in the suprapyramidal layer, compared to the adjacent *radiatum*, is well localized and notably, both areas contain the apical dendrites of CA3 pyramidal neurons, whereas the suprapyramidal zone contains proximal dendrites and stratum *radiatum* includes the distal portions. Moreover, the reduction observed in *radiatum*/LM (CA1) and *lucidum/radiatum* (CA3a) suggest that a relatively high BDNF-IR in distal dendrites may be a general characteristic of the hippocampal neurons in the VPA-model. Even though we cannot predict the functional consequence of the reduced BDNF-IR ratio, this result is in accordance with the proposed alteration in hippocampal circuitry.

Interestingly, the variation in BDNF-IR in the suprapyramidal layer and *stratum* *lucidum* may be related to the reduction in BDNF transcripts in the inferior blade of the DG. Thus, it is plausible that a reduction in BDNF mRNA in granule neurons may determine reduced BDNF peptide available for anterograde transport to mossy fiber terminals that target dendritic arbors of CA3 pyramidal neurons. However, we cannot precise whether the variation of BDNF-IR specifically occurs in mossy fiber terminals and/or in pyramidal neuron dendrites.

Reduction of long 3′UTR-containing BDNF transcripts in the inferior blade of the DG and in the polymorphic layer of the CA3 region may determine mBDNF/proBDNF balance. *L-bdnf* transcripts contain the 3′UTR segment necessary for dendritic targeting and synaptic activity-regulated translation (An et al., 2008), even though dendritic targeting and translation are not exclusive of *L-bdnf* (Baj et al., 2011; Vicario et al., 2015). Interestingly, evidence indicates that dendritic translation of *L-bdnf* essentially produces proBDNF, which promotes spine elimination through the binding to the low-affinity p75^NTR^ receptor, while a little portion is extracellularly cleaved to mBDNF and promotes synaptic spine maturation through the TrkB receptor activation (Orefice et al., 2013, 2016). Dendritic *L-bdnf* levels are undetectable in basal conditions by conventional ISH methods (Chiaruttini et al., 2009); however, in the present study, we observed variations of *L-bdnf* mRNA levels in the somatic area that can be considered as a global index of BDNF transcript availability. Hence, reduced *L-bdnf* levels in specific regions of the hippocampus, as we report in this work, may determine insufficient synaptic levels of proBDNF, thereby increasing the mBDNF/pro-BDNF ratio and, potentially affecting important developmental processes that occurring at juvenile age, such as circuit refinement.

Finally, the functional consequences of altered regional BDNF expression in the hippocampal network must be further analyzed, along with the proBDNF/mBDNF index, using specific antibodies. However, the increased memory index, in accordance with our previous report (Peralta et al., 2016), shows a distinctive hippocampal functioning in the VPA-treated rat. Interestingly, in addition to the core signs of autism, supplementary high order cognitive alterations have also been described in autism, as for instance: episodic memory difficulties but normal or enhanced memory for facts, percepts, skills, routines and associations (Boucher and Mayes, 2012).

In conclusion, our results support a BDNF region-specific downregulation in the juvenile hippocampus triggered by VPA exposure at early stages of brain development, suggesting an adaptive reduction of BDNF levels after VPA that can impair, but also improve, certain behavioral and cognitive performances. Thus, a comprehensive evaluation of the local changes of hippocampal BDNF isoforms in the VPA-model of autism is necessary to understand the genesis of the altered connectivity in the ASD brain.

## Data Availability Statement

The datasets generated for this study are available on request to the corresponding author.

## Ethics Statement

The animal study was reviewed and approved by Ethic Committee of the Chilean Science and Technology National Commission (CONICYT).

## Author Contributions

EA, JF, and AA participated in the design of the study, analysis and interpretation of data and wrote the article. KM-G participated in the analysis and interpretation of data and editing the article. CF, FP, and FA performed the experiments, analyzed the data and participated in the final correction of the article. All authors gave approval of the final version to be published, and agreed to be accountable for all aspects of the work.

## Conflict of Interest

The authors declare that the research was conducted in the absence of any commercial or financial relationships that could be construed as a potential conflict of interest.
